# Disease progression, treatments, hospitalization, and clinical outcomes in acute GVHD: a multicenter chart review

**DOI:** 10.1038/s41409-022-01764-w

**Published:** 2022-07-30

**Authors:** Shernan G. Holtan, Jingbo Yu, Hannah K. Choe, Dilan Paranagama, Jackson Tang, Ahmad Naim, John Galvin, H. Joachim Deeg

**Affiliations:** 1 Univeristy of Minnesota, Minneapolis, MN USA; 2 Incyte Corporation, Wilmington, DE USA; 3 The Ohio State University, Columbus, OH USA; 4 Asclepius Analytics, New York, NY USA; 5 University of Illinois Cancer Center, Chicago, IL USA; 6 Fred Hutchinson Cancer Research Center, Seattle, WA USA

**Keywords:** Bone marrow transplantation, Graft-versus-host disease

## Abstract

Acute graft-versus-host disease (GVHD) remains a barrier to successful allogeneic hematopoietic cell transplantation (HCT) outcomes. This multicenter, retrospective chart review describes disease progression, treatment patterns, hospitalizations, and clinical outcomes among 475 patients (≥12 years old) who developed grades II–IV acute GVHD after their first HCT (January 2014–June 2016). Median (interquartile range) age at HCT was 55 (44–63) years. From the date of acute GVHD diagnosis, 190 patients (40.0%) experienced progression to more severe disease and/or developed new organ involvement. Among 431 patients with grades II–IV acute GVHD at diagnosis, 73.1% received first-line systemic corticosteroids. During follow-up (median 524 days since acute GVHD diagnosis), 23.4% of patients had an increase in steroid dose and 44.4% were unable to taper below 10 mg/day. Over half of patients (54.9%) required ≥1 hospital readmission within 100 days post-HCT (≥2 readmissions in 22.3%); mean inpatient length of stay upon readmission was 27.5 days. Approximately half of patients (52.8%) died during follow-up; 1-year overall mortality from date of acute GVHD diagnosis and nonrelapse mortality rates were 35.2% and 25.5%, respectively. Overall, patients who developed acute GVHD following HCT had poor clinical outcomes, highlighting the unmet need for early and effective treatment strategies.

## Introduction

Allogeneic hematopoietic cell transplantation (HCT) is a potentially curative treatment option for several hematologic malignancies and nonmalignant blood disorders [1, 2]. However, the clinical course following an allogeneic HCT can be complicated by acute graft-versus-host disease (GVHD), which has a significant effect on morbidity and mortality [3, 4]. Approximately 30–60% of patients who undergo an allogeneic HCT develop acute GVHD [5–8].

The standard first-line therapy for grades II to IV acute GVHD is immunosuppression with systemic corticosteroids, which are effective in approximately 35% to 60% of patients [9–11]. Patients who do not achieve a sustained complete response to first-line therapy tend to experience acute GVHD progression, increasing the risk of mortality [3]. GVHD grade at diagnosis has been shown to correlate with overall survival [12].

Our current understanding of acute GVHD is based largely on registry studies [13, 14] and may not include data regarding the management and clinical outcomes of acute GVHD. The aim of this study was to update our understanding of acute GVHD in the setting of current practices in conditioning regimens, stem cell sources, and GVHD prophylaxis. The specific objective of this multicenter retrospective chart review was to describe the real-world picture of clinical courses, treatments, hospitalization rates, and outcomes of patients who developed acute GVHD after HCT in the era immediately before multicenter clinical trial testing and subsequent approval of ruxolitinib by the US Food and Drug Administration for steroid-refractory acute GVHD.

## Methods

### Study design and patients

A multicenter, retrospective chart review was conducted at 11 large academic and community transplant centers in the United States for patients who received allogeneic HCT between January 1, 2014, and June 30, 2016 (study period). Participating sites conducted ≥50 adult allogeneic HCTs and diagnosed and treated ≥20 patients for acute GVHD during the study period. Institutional review board approval was obtained from participating institutions.

Physicians were asked to review individual electronic medical records for patients aged ≥12 years who had undergone their first allogeneic HCT during the study period and subsequently developed grades II to IV acute GVHD (per Center for International Blood and Marrow Transplant Research criteria) at any time during the follow-up period (ie, transplant to the end of data availability or death). Diagnoses were made based on physician discretion, including the decision to perform histology evaluation, and which tissue to biopsy. Exclusion criteria included >1 allogeneic HCT, participation in a trial for GVHD prophylaxis during the study period (participation in a GVHD treatment trial was permitted), use of Janus kinase (JAK) inhibitors for any condition, and the inability to disclose complete GVHD-related medical history for any reason (Table 1).

**Table 1:** Inclusion and exclusion criteria.

Inclusion criteria
• Aged 12 years and older
• Received allogeneic HCT between January 1, 2014 and June 30, 2016 from any donor source using bone marrow, peripheral blood stem cells, or cord blood
• Diagnosed with IBMTR Severity Index II-IV acute GVHD (early onset acute, classical acute, or late onset acute) any time after transplant to end of data availability, or patient death
**Exclusion Criteria**
• >1 allogeneic HCT
• Participated in GVHD prophylaxis clinical trial during the study period (participation in GVHD treatment trials is allowed)
• Patients treated with any JAK inhibitors for any condition
• Patients who may not disclose their entire GVHD-related medical history due to any obligations

*GVHD* graft-versus-host disease, *HCT* hematopoietic cell transplantation, *IBMTR* International Blood and Marrow Transplant Research, *JAK* Janus kinase.

### Data Collection

De-identified patient data were collected through an electronic case report form between December 2017 and January 2019. Site physicians were responsible for review of the medical records of eligible patients. Eligible patients were observed retrospectively from the date of allogeneic HCT to end of most recent follow-up or death. At each participating site, patients were sampled based on transplant date, beginning with the most recent transplant recipients and continuing sequentially until a sufficient number of patient records were acquired or data collection closed. Consecutive patients were reviewed and data for eligible patients were collected during the data collection time frame based on site capability. The targeted number for study inclusion was 500 patients. Data collected included patient demographics and clinical characteristics, acute GVHD grade and organ involvement at diagnosis, changes in acute GVHD grade or organs involved, acute GVHD recurrence, treatment regimens, inpatient resource utilization, all-cause mortality, and 12-month overall mortality and nonrelapse mortality (NRM). For this analysis, corticosteroid refractoriness was defined as requiring ≥1 additional systemic GVHD therapy, and corticosteroid dependence was defined as the inability to taper high-dose corticosteroids [≥1 mg/kg] by ≥25% or to <10 mg/day. NRM was defined as experiencing death and never having a relapse of underlying disease at follow-up.

### Statistical analyses

Frequencies and percentages were reported for categorical variables; mean, SD, median, and interquartile range (IQR) values were determined for continuous variables. Twelve-month overall mortality and NRM were calculated using Kaplan–Meier estimates; patients were censored at the end of the follow-up period.

## Results

### Patient demographics and clinical characteristics

Data were collected for 475 patients who met study inclusion criteria from 11 transplant centers (listed in Acknowledgments); median (IQR) age at HCT was 55 [44–63] years, and 57.1% of patients were male (Table 2). The most common underlying malignancies were acute myeloid leukemia (38.7%), acute lymphoid leukemia (15.8%), and myelodysplastic syndrome (14.9%). The main HCT graft source was peripheral blood (67.6%), and transplant conditioning was most commonly conducted using myeloablative regimens (47.2%). The majority of GVHD-prophylaxis consisted of a calcineurin inhibitor (tacrolimus or cyclosporine A), and at least one additional immunosuppressive agent (methotrexate, mycophenolate, sirolimus or antithymocyte globulin). Median (IQR) time from transplant to acute GVHD diagnosis was 32 [22–48] days. Among all 475 patients at diagnosis, the most common tissues biopsied and evaluated by histology skin (33.9%), upper GI (33.9%), and lower GI (33.1%). Liver biopsies were less frequent (2.1%). Median (IQR) follow-up from acute GVHD diagnosis to death/last visit was 524 (165–954) days.

**Table 2:** Patient demographics and baseline clinical characteristics.

	Acute GVHD (*N* = 475)
Age, years, median (IQR)	55 [44–63]
Male, *n* (%)	271 (57.1)
White, *n* (%)	409 (86.1)
Insurance status at transplant,^a^ *n* (%)	
Private or group health insurance	350 (73.7)
Medicare	106 (22.3)
Medicaid	40 (8.4)
Other^b^	24 (5.1)
Underlying malignancy, *n* (%)	
Acute myeloid leukemia	184 (38.7)
Acute lymphoid leukemia	75 (15.8)
Myelodysplastic syndrome	71 (14.9)
Non-Hodgkin lymphoma	36 (7.6)
Chronic myeloid leukemia	24 (5.1)
Multiple myeloma	23 (4.8)
Other^c^	62 (13.1)
Remission status of primary disease at transplant, *n* (%)	
Complete remission	309 (65.1)
Stable disease	59 (12.4)
Partial remission	48 (10.1)
Progressive disease	26 (5.5)
Untreated	3 (0.6)
Not assessed	30 (6.3)
HCT comorbidity index at transplant, *n* (%)	
Low risk (0)	88 (18.5)
Intermediate risk (1–2)	144 (30.3)
High risk (≥3)	230 (48.4)
Unknown	13 (2.7)
Year of transplant, *n* (%)	
2014	135 (28.4)
2015	239 (50.3)
2016	101 (21.3)
Transplant setting,^d^ *n* (%)	
Inpatient	439 (92.4)
Outpatient	31 (6.5)
Graft source, *n* (%)	
Peripheral blood	321 (67.6)
Umbilical cord blood	80 (16.8)
Bone marrow	69 (14.5)
Unknown	5 (1.1)
HLA donor type, *n* (%)	
Matched–unrelated	212 (44.6)
Matched–related	108 (22.7)
Mismatched–unrelated	101 (21.3)
Mismatched–related	51 (10.7)
Unknown	3 (0.6)
Method used to determine degree of HLA match, *n* (%)	
Out of 6	87 (18.3)
Out of 8	126 (26.5)
Out of 10	262 (55.2)
Transplant conditioning regimen, *n* (%)	
Myeloablative	224 (47.2)
Reduced intensity	134 (28.2)
Nonmyeloablative	117 (24.6)
GVHD prophylaxis therapy,^e^ *n* (%)	
Tacrolimus-based	268 (56.4)
Mycophenolate	209 (44.0)
Methotrexate	172 (36.2)
Cyclosporine-based	124 (26.1)
Antithymocyte globulin	37 (7.8)
High-dose cyclophosphamide (posttransplant)	27 (5.7)
Sirolimus	20 (4.2)
Other	22 (4.6)
Duration of follow-up,^f^ days, mean (SD)	718.1 (478.1)

*GVHD* graft-versus-host disease, *HCT* hematopoietic cell transplantation, *HLA* human leukocyte antigen, *IQR* interquartile range.

^a^Insurance status was not available for 1 patient; 48 patients (10.1%) had multiple types of insurance coverage; 2 patients (0.4%) were uninsured.

^b^Includes government-sponsored Veterans Affairs/military (*n* = 7 [1.5%]), employer-sponsored disability insurance (*n* = 3 [0.6%]), and other (*n* = 14 [2.9%]).

^c^“Other” primary diseases specified in ≥2 patients included Hodgkin lymphoma and aplastic anemia (*n* = 7 each); myeloproliferative disease (*n* = 6); and sickle cell anemia (*n* = 2).

^d^Transplant setting was unknown for 5 patients.

^e^Patients may have used >1 GVHD prophylaxis therapy.

^f^Patients were followed up for ≥2 years from transplant until death or end of observation, whichever occurred first.

### Disease progression

**Fig. 1:**
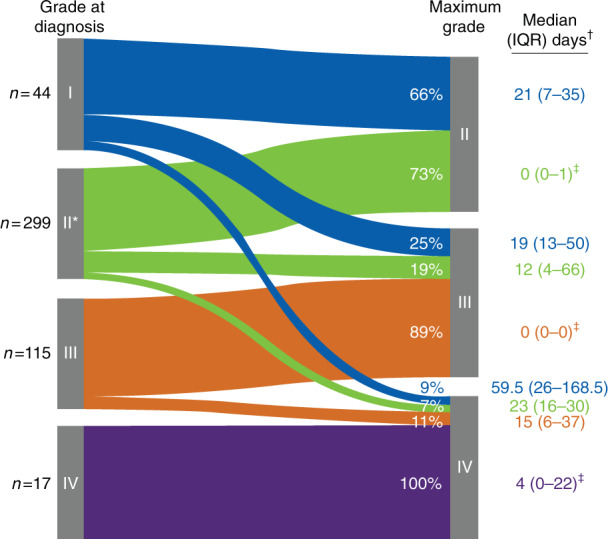
Acute GVHD severity at diagnosis and maximum grade. GVHD graft-versus-host disease, IQR interquartile range. *Maximum grade was unknown for 1 patient who had grade II acute GVHD at diagnosis. ^†^Time from acute GVHD diagnosis to maximum grade. ^‡^Patients may have remained at the same grade from diagnosis to maximum grade, but their disease progressed with new organ involved.

**Fig. 2:**
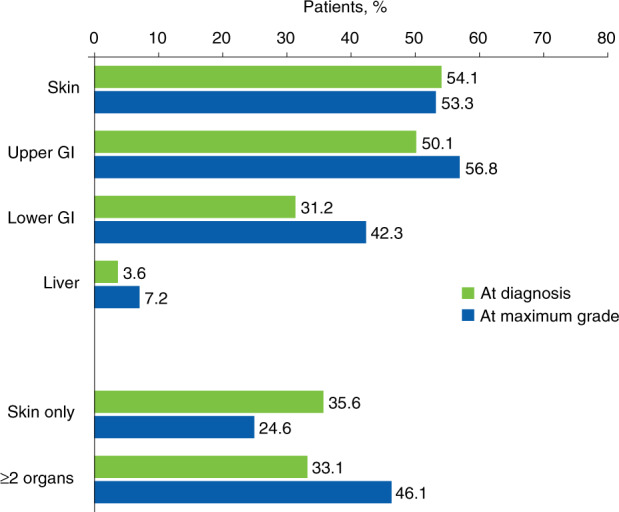
Acute GVHD organs involved* at diagnosis and maximum grade. GI gastrointestinal tract, GVHD graft-versus-host disease. *“Skin only” includes patients who only had skin involvement; “skin” includes patients who had skin involvement with/without acute GVHD in other organs.

At the time of acute GVHD diagnosis, 299 patients (62.9%) had grade II acute GVHD (Fig. 1). From the date of diagnosis, 40.0% of patients had an increase in acute GVHD grade or developed new organ involvement. Median (IQR) time from diagnosis to maximum grade was 19 [5–48] days. At maximum acute GVHD grade, 248 patients (52.2%) had grade II disease, and 226 patients (47.6%) had grades III or IV disease. Among 169 patients who had only skin involvement at diagnosis, 30.8% developed acute GVHD in other organs during progression of acute GVHD to maximum grade. There was also an increase in the proportion of patients who had lower gastrointestinal involvement (31.2–42.3%) and ≥2 organs involved (33.1–46.1%) during acute GVHD progression (Fig. 2).

### Treatment patterns

All 475 patients who met inclusion criteria received treatment. Among 431 patients with grades II to IV acute GVHD at time of diagnosis, 97.9% received corticosteroids as first-line treatment. Of these, 73.1% received systemic corticosteroids and 26.9% received non-systemic steroids (topical or non-absorbable); 49.0% of patients initiated systemic corticosteroids on the day of diagnosis. Median (IQR) time from diagnosis to initiation of systemic corticosteroids was 0 (0–2) days. Median starting dose for patients receiving oral or intravenous systemic corticosteroids as first-line therapy (*n* = 314) was 79 mg/day (1.0 mg/kg) for prednisone and 190 mg/day (2.1 mg/kg) for methylprednisolone. Among 138 patients who received prednisone as the first-line therapy, the starting dose was ≤1.0 mg/kg in 91 patients (65.9%; acute GVHD grade at diagnosis among the 91 patients: I, 9.9%; II, 75.8%; III, 13.2%; IV, 1.1%). Of 189 patients treated with first-line methylprednisolone, the starting dose was ≤2.0 mg/kg in 162 patients (85.7%; acute GVHD grade at diagnosis among the 162 patients: I, 1.2%; II, 59.9%; III, 30.9%; IV, 8.0%).

During the follow-up period (time from GVHD diagnosis to end of data availability; median, 524 days), 23.4% of patients had an increase in steroid dose; 44.4% were unable to taper below 10 mg/day. Among patients who were dependent on or refractory to corticosteroids (*n* = 55 [11.6%] and *n* = 113 [23.8%], respectively); 87.5% were unable to taper below 10 mg/day, and 36.3% had an increase in steroid therapy (detailed analysis reported elsewhere) [15]. Additional systemic anti-GVHD therapy was received by 89 of these patients (53.0%), most frequently sirolimus (19.1%), polyclonal antibodies (18.0%), mycophenolate mofetil (16.9%), tocilizumab (15.7%), extracorporeal photopheresis (13.5%), and etanercept (13.5%). Of these patients, 41.6% had an increase in corticosteroid dose before receiving additional therapy, and 25.8% used ≥2 additional therapies. Median (IQR) time from corticosteroid initiation to additional therapy was 21 [8–32] days.

### Clinical outcomes and survival

**Fig. 3:**
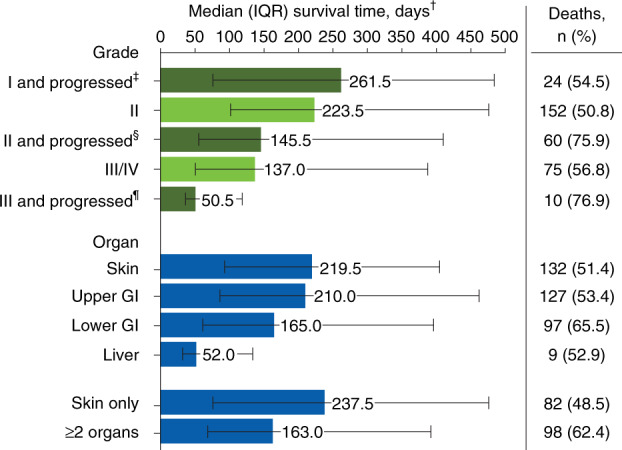
Patient deaths by acute GVHD severity and organs* involved at diagnosis. GI gastrointestinal tract, GVHD graft-versus-host disease, IQR interquartile range. *Organ stage 1–4 was considered organ involvement; patients could have multiple organs involved. ^†^Time from acute GVHD diagnosis to death. ^‡^Patients who had grade I acute GVHD at diagnosis but whose disease progressed to a higher grade later. ^§^A subset of patients who had grade II acute GVHD at diagnosis but whose disease progressed to a higher grade later. ^¶^A subset of patients who had grade III acute GVHD at diagnosis but whose disease progressed to a higher grade later.

Acute GVHD recurred in 41.9% of patients during follow-up (mean [SD] time from full response to first recurrence, 73.4 [78.6] days); recurrence was managed by increasing the corticosteroid dose in 51.3% of patients. During follow-up, 191 patients (40.2%) developed chronic GVHD; the mouth (50.3%) and skin (46.1%) were most commonly involved at diagnosis of chronic GVHD. Almost a quarter (23.2%) of patients experienced a relapse of their underlying malignancy during follow-up. Patients who developed grades III/IV acute GVHD during their initial HCT hospital stay (*n* = 43) had a longer median (IQR) length of stay (43 [33–64] days) compared with patients diagnosed with grade II acute GVHD during HCT hospitalization (*n* = 83; 37 [31–50] days). Among all 475 patients, ≥1 hospital readmission was required by 54.9% of patients within 100 days post-HCT; mean (SD) inpatient length of stay upon readmission was 27.5 (192.3) days. The mean (SD) number of hospital readmissions per patient was 1.6 (0.8), and 22.3% of patients had ≥2 readmissions. The mean (SD) number of intensive care unit admissions per patient was 0.2 (0.4). Infection within 100 days post-HCT occurred in 45.3% of patients and most commonly included viral (35.8%), bacterial (20.8%), and fungal (2.9%) infections. Twelve-month overall mortality from the time of acute GVHD diagnosis and NRM rates were 35.2% and 25.5%, respectively. Patients with grade II acute GVHD at diagnosis had lower 12-month mortality rates than those with grades III or IV acute GVHD at diagnosis (overall mortality, 31.8% vs 41.7%; NRM, 23.4% vs 29.5%). Half of patients (251/475 [52.8%]) died at a median (IQR) of 213 (77–430) days after acute GVHD diagnosis. The most common primary causes of death were primary disease (33.1%), infection (13.1%), acute GVHD (10.8%), and organ failure (10.8%). Mortality rates during follow-up (Fig. 3) were increased among patients who had higher-grade acute GVHD and among patients who progressed in grade from diagnosis (grade II, 50.8% [152/299]; grades III/IV, 56.8% [75/132]; grade II and progressed, 75.9% [60/79]; grade III and progressed, 76.9% [10/13]).

## Discussion

In this multicenter, retrospective chart review, 40% of patients with acute GVHD experienced disease progression and developed severe disease (grades III–IV) despite the use of systemic treatment. Survival was poor, with approximately half (53%) of patients dying within 7 months of acute GVHD onset, and 1-year overall mortality from date of acute GVHD diagnosis and NRM rates were 35% and 26%, respectively.

In this study period, (2014–2016), more than half of patients (55%) required ≥1 hospital readmission at any time during follow-up, including an average of 0.2 intensive care unit admissions per patient. GVHD grade correlated with overall survival; although patients with grades III and IV acute GVHD had the worst outcomes, patients with grade II at onset also had high mortality rates (51%). These findings are largely consistent with previous reports. A retrospective review of a hospital discharge database (study period, 2011–2016) showed high hospital readmission rates (78%) and inpatient mortality rates (20%) within 100 days post-HCT [16]. Another retrospective analysis of hospital readmissions following allogeneic HCT (study period, 2006–2009) reported high hospital readmission rates (86%) and 2-year mortality rates (58%) among patients diagnosed with acute GVHD; mortality rates were higher among patients with grades III and IV versus grades I and II GVHD (86% vs 45%) [17]. Although results are not directly comparable, they suggest that hospital readmission rates remain high for patients with acute GVHD and support an increase in mortality rates with time following acute GVHD diagnosis [13]. The acute GVHD relapse rate of 23% and overall survival rate of 65% in the present study are comparable to trends reported in other retrospective registry analyses [13, 14].

Limitations to this study include the retrospective nature of the analysis and possible differences between the various study centers in assessing GVHD (eg, potentially varied criteria for diagnosis), documenting data, and cause of death attribution. As patients with steroid-refractory aGvHD usually die with infection and multi-organ failure [18], it can be difficult to ascertain whether the cause of death was aGVHD directly, or a secondary and associated cause. The use of JAK inhibitors was an exclusion criterion for this analysis (study period January 2014–June 2016). The JAK1/JAK2 inhibitor ruxolitinib was approved by the US Food and Drug Administration for the treatment of steroid-refractory acute GVHD in mid-2019 [19]. While there is no standard second-line treatment for acute GVHD [20], it will be important to explore changes in the management and outcomes of acute GVHD with availability of newer treatment options.

In conclusion, these findings suggest that there is a need for effective and tolerable treatments administered early in the disease course of acute GVHD to prevent or reverse disease progression.

## Data Availability

Access to individual patient-level data is not available for this study. Information on Incyte’s clinical trial data sharing policy and instructions for submitting clinical trial data requests are available at: https://www.incyte.com/Portals/0/Assets/Compliance%20and%20Transparency/clinical-trial-data-sharing.pdf?ver=2020-05-21-132838-960.
