# Correction to: Disease progression, treatments, hospitalization, and clinical outcomes in acute GVHD: a multicenter chart review

**DOI:** 10.1038/s41409-022-01857-6

**Published:** 2022-11-11

**Authors:** Shernan G. Holtan, Jingbo Yu, Hannah K. Choe, Dilan Paranagama, Jackson Tang, Ahmad Naim, John Galvin, H. Joachim Deeg

**Affiliations:** 1 Univeristy of Minnesota, Minneapolis, MN USA; 2 Incyte Corporation, Wilmington, DE USA; 3 The Ohio State University, Columbus, OH USA; 4 Asclepius Analytics, New York, NY USA; 5 University of Illinois Cancer Center, Chicago, IL USA; 6 Fred Hutchinson Cancer Research Center, Seattle, WA USA

**Keywords:** Bone marrow transplantation, Graft-versus-host disease

Correction to: *Bone Marrow Transplantation* (2022) 57:1581–1585; 10.1038/s41409-022-01764-w, published online 30 July 2022

The article “Disease progression, treatments, hospitalization, and clinical outcomes in acute GVHD: a multicenter chart review”, written by Shernan G. Holtan, Jingbo Yu, Hannah K. Choe, Dilan Paranagama, Jackson Tang, Ahmad Naim, John Galvin and H. Joachim Deeg., was originally published Online First without Open Access. After publication in volume 57, issue 10, page 1581–1585 the author decided to opt for Open Choice and to make the article an Open Access publication. Therefore, the copyright of the article has been changed to © The Author(s) 2022 and the article is forthwith distributed under a Creative Commons Attribution 4.0 International License, which permits use, sharing, adaptation, distribution and reproduction in any medium or format, as long as you give appropriate credit to the original author(s) and the source, provide a link to the Creative Commons licence, and indicate if changes were made. The images or other third party material in this article are included in the article’s Creative Commons licence, unless indicated otherwise in a credit line to the material. If material is not included in the article’s Creative Commons licence and your intended use is not permitted by statutory regulation or exceeds the permitted use, you will need to obtain permission directly from the copyright holder. To view a copy of this licence, visit http://creativecommons.org/licenses/by/4.0.

